# Analysis of the DNA methylation level of cancer-related genes in colorectal cancer and the surrounding normal mucosa

**DOI:** 10.1186/s13148-017-0352-4

**Published:** 2017-05-18

**Authors:** Tamotsu Sugai, Masakazu Yoshida, Makoto Eizuka, Noriyuki Uesugii, Wataru Habano, Kouki Otsuka, Akira Sasaki, Eiichiro Yamamoto, Takayuki Matsumoto, Hiromu Suzuki

**Affiliations:** 1 Department of Molecular Diagnostic Pathology, School of Medicine, Iwate Medical University, 19-1, Morioka, 020-8505 Japan; 2 Department of Surgery, School of Medicine, Iwate Medical University, 19-1, Morioka, 020-8505 Japan; 3 Department of Pharmacodynamics and Molecular Genetics, School of Pharmacy, Iwate Medical University, 19-1, Morioka, 020-8505 Japan; 4 Department of Molecular Biology, Sapporo Medical University, Chuo-ku, Sapporo, 060-8556 Japan; 5 Department of Internal Medicine, Division of Gastrointestinal Tract, School of Medicine, Iwate Medical University, 19-1, Morioka, 020-8505 Japan

**Keywords:** Colorectal cancer, Crypt isolation, DNA methylation, Normal mucosa, Microsatellite instability

## Abstract

**Background:**

Two molecular pathways promote the development of colorectal cancer (CRC). One is termed “microsatellite stable” (MSS) whereas the other is characterized by “microsatellite instability” (MSI or MIN). In addition, the CpG island methylation phenotype is known to be an important alteration as a third molecular type. Thus, DNA methylation is thought to provide potential biomarkers for assessment of cancer risk in normal mucosa. In addition, it is also known that colonic location is an important parameter in the development of CRC.

**Methods:**

We examined the surrounding normal mucosa in three parts of the colon. Next, we quantified DNA methylation levels of *SFRP1*, *SFRP2*, *SFRP5*, *DKK2*, *DKK3*, *mir34b/c*, *RASSF1A*, *IGFBP7*, *CDKN2A*, and *MLH1* in isolated cancerous glands and crypts of normal colorectal mucosa adjacent to CRCs using a pyrosequencer.

**Results:**

DNA methylation levels of *SFRP1*, *SFRP2*, *DKK2*, and *mir34b/c* were significantly higher in CRCs with an MSS phenotype than in those with an MSI phenotype. The average level of methylation in normal crypts did not decrease with the distance from the tumor, irrespective of microsatellite status or the tumor location. DNA methylation levels in *SFRP1* and *SFRP2* genes in normal crypts were significantly higher in left-side than right-side CRC with an MSS phenotype. Finally, the genes were classified into three types based on the methylation frequencies in normal crypts, including type I (*SFRP1* and *SFRP2I)*, type II (*DKK2* and *mir34b/c*), and type III (others).

**Conclusions:**

Our results showed that DNA methylation of *SFRP1* and *SFRP2* might be useful to predict cancer risk of surrounding normal mucosa. In addition, a field effect may be present in CRC, affecting both adjacent and non-adjacent normal mucosa.

**Electronic supplementary material:**

The online version of this article (doi:10.1186/s13148-017-0352-4) contains supplementary material, which is available to authorized users.

## Background

Colorectal cancer (CRC) develops via accumulation of numerous genetic and epigenetic alterations, as normal colorectal epithelium transforms into an adenoma, then progresses to cancer [1, 2]. In this model, CRC is caused by alterations in the Wnt and TGF-β signaling pathways, activation of *KRAS*, and inactivation of *APC* and *TP53* [2, 3]. Most CRCs (80–90%) can be explained by this mechanism [2, 3]. On the other hand, 10–20% of CRCs are characterized by a defective mismatch repair system, resulting in increased genetic mutations and microsatellite instability (MSI) [2, 3]. Previous study has identified two types of CRC: a chromosomal instability (CIN) type (also termed microsatellite stable (MSS)) and a type with microsatellite instability (MIN and MSI) [4]. Whereas the former is closely associated with multiple chromosomal alterations and mutation of *TP53*, the latter is characterized by MSI, CpG island methylation on a genome-wide level (CpG island methylator phenotype (CIMP)), and mutations in *BRAF* [4, 5]. Therefore, it is thought that MSS (CIN) and MSI types of cancers are mutually exclusive in terms of molecular pathways [2–4]. DNA methylation is a third potential mechanism underlying CRC initiation, as it epigenetically silences genetic expression and contributes to the onset and development of CRC [6–8]. In particular, it is well known that genome-wide DNA methylation plays an important role in the development of specific types of CRC (MIN type). Recent studies have revealed integrated molecular and transcriptomic patterns in CRC, including new insights from the Consensus Molecular Subtype (CMS) Consortium. Molecular classification of CMS is based on both expression and genetic patterns [CMS 1, 2, 3, 4, and mixed features] [9–11]. In addition, new classification systems have been proposed based on comparisons of gene expression levels in tumor cells with those in corresponding normal cell populations (stem-like, inflammatory, transit-amplifying, goblet-like cells and enterocytes). Although these molecular classifications are useful to elucidate colorectal carcinogenesis, dichotomization of molecular classification (e.g., MSS and MSI) has been useful in that dichotomization shows mutually exclusive phenomena.

It is well known that tumor location (left side versus right side of the colon) affects colorectal carcinogenesis [12, 13]. Previous study has shown that CRCs with an MSI phenotype with CIMP-high status, infrequent *TP53* mutation, and frequent *BRAF* mutations preferentially occur on the right side of the colon. In contrast, CRC with an MSS phenotype that is characterized by CIMP-low status, *TP53* mutation, and frequent copy number alterations is commonly found on the left side of the colon. These findings suggest that tumor location may be associated with the development of CRC.

The concept of field effects in cancer was introduced to explain the development of multiple primary tumors, local recurrence and abnormal tissue surrounding cancer tissue, and the presence of multiple precancerous lesions [14–16]. This concept is based on the susceptibility of normal mucosa to undergoing early molecular changes that lead to the development of CRC [14–16]. Recent studies have shown that DNA methylation of cancer-related genes is found in the normal mucosa surrounding the cancer. This finding suggests that precancerous cells adjacent to the tumor may appear to be histologically normal but can harbor some, but not all, of the DNA methylations that are seen in fully developed tumors [17–20].

The Wnt signal pathway plays an important role in early colorectal carcinogenesis [21]. In addition, Wnt signal-related genes are frequently methylated in colorectal tumors, including *SFRP* and *DKK* [22, 23]. It is well known that *RASSF1A* and *mir34b/c* are also methylated in early colorectal carcinogenesis, such as colorectal adenoma [24, 25]. On the other hand, CRC with an MSI phenotype is characterized by DNA methylation of *MLH1*, *IGFBP7*, and *CDKN2A* genes [26–28]. However, important points should be taken into consideration when selecting methylation markers [7]. It is well known that DNA methylation is influenced by various factors, including aging and environmental factors [7, 29]. According to a previous study [7], there appear to be two types of methylation that are associated with cancer progression: type A (for age-related) methylation and type C (for cancer-specific) methylation [7]. Although the initial report showed that type A methylation arises as a function of age in normal colorectal epithelial cells, such methylation may result in a state that predisposes the patient for tumor formation in the colon [30]. Thus, assessment of DNA methylation levels of genes expressed in normal cryptal cells, including *SFRP1*, *SFRP2*, *SFRP5*, *DKK2*, *DKK3*, *mir34b/c*, *RASSF1A*, *IGFBP7*, *CDKN2A*, and *MLH1* might be useful in early detection or risk prediction of CRC.

Here, we used a crypt isolation method to assess the levels of epigenetic events in cancerous and normal crypts. In this way, we examined how epigenetic events, MSI status, and tumor location could be involved in field cancerization. We studied the DNA methylation status of specific loci, including *SFRP1*, *SFRP2*, *SFRP5*, *DKK2*, *DKK3*, *mir34b/c*, *RASSF1A*, *IGFBP7*, *CDKN2A*, and *MLH1.* In this way, we determined their involvement in CRC development in isolated normal and cancerous crypts.

## Methods

### Patients

The study focused on patients who underwent curative surgery for colorectal cancer (CRC) at Iwate Medical University Hospital from January 2011 to December 2012. Participants included a non-consecutive series of 100 patients for whom medical records and the pathological examination were complete. Patients who underwent preoperative chemoradiotherapy or emergency surgery and patients who had evidence of hereditary non-polyposis colorectal cancer or familial adenomatous polyposis were excluded from the study. In addition, only primary CRCs were included. The clinicopathological parameters of the patients were confirmed by reviewing the patient medical records and pathology files.

The pathological diagnosis and staging were performed according to the Classification of the Japanese Society for Cancer of the Colon and Rectum [31]. Tumor locations were noted as left sided (rectum, sigmoid, and descending) or right sided (transverse, ascending, and cecum). Clinicopathological findings based on tumor location (left, rectum, sigmoid, and descending colon; right, transverse, ascending, and cecum) are summarized in Table 1.

**Table 1:** Clinicopathological findings in colorectal cancers and their locations

	Total	Left-sided CRC	Right-sided CRC
	100	74 (%)	26 (%)
Median (age)	69.9 (36–88)	68.3 (36–88)	73.1 (47–84)
Sex (man/woman)	59/41	44/31	15/11
Location			
Cecum	9	–	9 (34.6)
Ascending	9	–	9 (34.6)
Transverse	8	–	8 (30.8)
Descending	7	7 (9.5)	–
Sigmoid	22	22 (29.7)	–
Rectum	45	45 (60.8)	–
Histology			
Differentiated-type	94	71 (96.0)	23 (88.5)
Undifferentiated-type	6	3 (4.1)	3 (11.5)
Dukes’ classification			
A	15	14 (18.9)	1 (3.9)
B	39	26 (35.1)	13 (50.0)
C	39	28 (37.8)	11 (42.3)
D	7	6 (8.1)	1 (3.8)

Informed consent was obtained from each subject according to the institutional guidelines, and the research protocols were approved by the ethics committee of Iwate Medical University Hospital.

### Tissue sampling

**Fig. 1:**
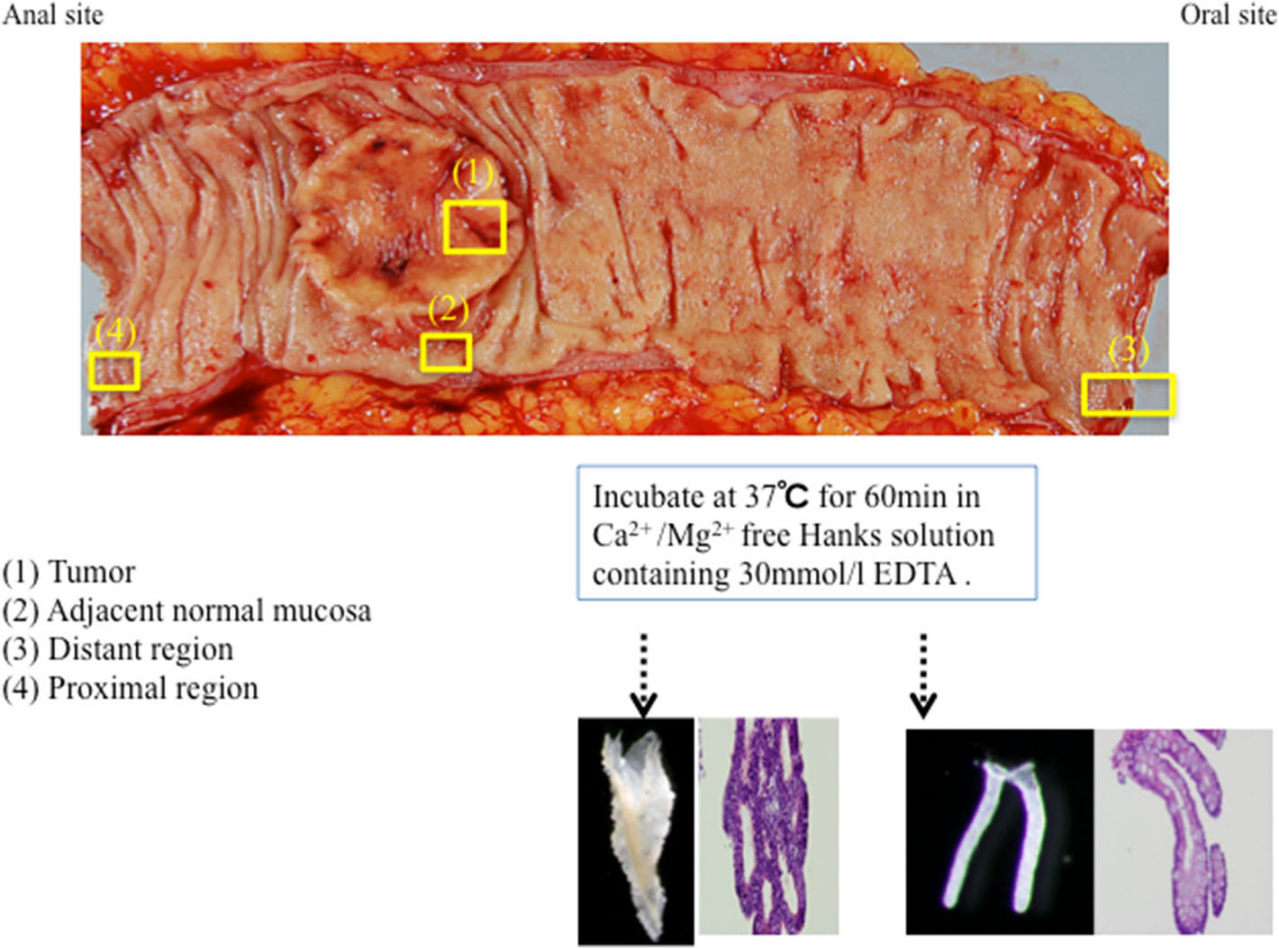
Scheme of the sample using a crypt isolation method

Normal colonic mucosa was obtained from three regions of the resected colonic mucosa, including the proximal margin, the region adjacent to the cancer, and the distal margin. The range of distances to the distal margin was between 5 and 17.6 cm (median, 8.7 cm). In addition, the distance from the proximal region to the cancer was between 3.5 and 11.4 cm (median 6.5 cm). The distance of the normal region adjacent to the cancer was within 1.5 cm. The sections were histologically normal based on routine examination. Cancer tissue was obtained from the central area of the tumor. Finally, the sampling method is illustrated in Fig. 1.

### Crypt isolation method and DNA extraction

Crypt isolation from tumors and three regions of normal colonic mucosa was performed in accordance with a previously reported method in order to obtain pure glands [32]. The isolated glands were processed routinely to confirm their nature using paraffin-embedded histological sections. Contamination by other tissues such as interstitial cells was not evident in the samples that were examined, as described in previous reports.

DNA from the tumor and from corresponding normal crypts was extracted by standard SDS proteinase K treatment. Samples were resuspended in TE buffer [10 mM Tris-HCl, 1 mM EDTA (pH 8.0)] to the equivalent of 1000 cells/μL.

### Analysis of microsatellite instability

We examined the microsatellite instability (MSI) status of colorectal tumors using five NCI markers, including BAT25, BAT26, D2S123, D5S346, and D17S250. MSI-high (MSI-H) was defined as two or more markers being unstable whereas MSI-low (MSI-L) was defined as one marker being unstable, and microsatellite stable (MSS) was defined as the absence of instability [33]. Normal alleles were typically represented by a major peak accompanied by a few minor peaks. The mobility shift of PCR products from tumor DNA was compared to that obtained from corresponding non-neoplastic crypts. MSI-low was included in MSS status in the present study.

### Pyrosequencing of DNA methylation

Assays to assess the methylation status of CpG dinucleotides in the loci of interest were designed using the PyroMark Assay Design 2.0 software (Qiagen, Inc.) [34]. For *SFRP1*, S*FRP2*, *SFRP5*, *DKK2*, *DKK3*, *mir34b/c*, *RASSF1A*, *IGFBP7*, *CDKN2A*, and *MLH1*, primers were designed to assess sequential CpG dinucleotides located in CpG islands near the transcription start site of each respective gene. The sequences of primers used here are listed in Additional file 1: Table S1. Following PCR amplification using the Qiagen PyroMark PCR kit (Qiagen #978703), the final biotin-labeled PCR product was purified, sequenced, and analyzed using the PyroMark Q24 MDx machine. The accuracy of the assays was confirmed using a series of known standard DNA samples before assessing the test samples.

#### Mutation analysis of the KRAS and BRAF genes

Mutations of exon 2 of the *KRAS* gene and exon 15 of the *BRAF* gene (V600E) were examined by pyrosequencing of tumor gland DNA samples. The methods for pyrosequencing of *KRAS* and *BRAF* were described previously [34].

### Statistical analysis

Differences in the levels of DNA methylation between groups were analyzed using Kruskal-Wallis tests (PRISM6; GraphPad software, La Jolla, CA, USA). If statistical differences between each group were found, statistical analysis of the two groups was further performed using Mann-Whitney *U* tests (PRISM6; GraphPad software, La Jolla, CA, USA) with a Bonferroni correction*.* Probabilities less than 0.05 were regarded as significant.

## Results

### Analysis of microsatellite instability in colorectal cancer

Out of the 100 CRC we examined, 6 tumors were classified as MSI-high, whereas 94 tumors were assigned into the MSI-negative/low category.

### DNA methylation levels in cancerous crypts compared to surrounding normal crypts based on MSI status

We examined DNA methylation levels of *SFRP1*, *SFRP2*, *SFRP5*, *DKK2*, *DKK3*, *mir34b/c*, *RASSF1A*, *IGFBP7*, *CDKN2A*, and *MLH1* genes in cancerous crypts isolated from MSS and MSI CRCs. In addition, we investigated DNA methylation levels in normal crypts from the surrounding normal mucosa in MSS and MSI CRCs. The DNA methylation levels of *SFRP1*, *SFRP2*, *DKK2*, and *mir34b/c* were significantly higher in MSS cancerous crypts than in those with the MSI phenotype. On the other hand, methylation levels of *MLH1* and *IGFBP7* markers in MSI phenotype cancerous crypts were higher than those of the MSS phenotype.

**Fig. 2:**
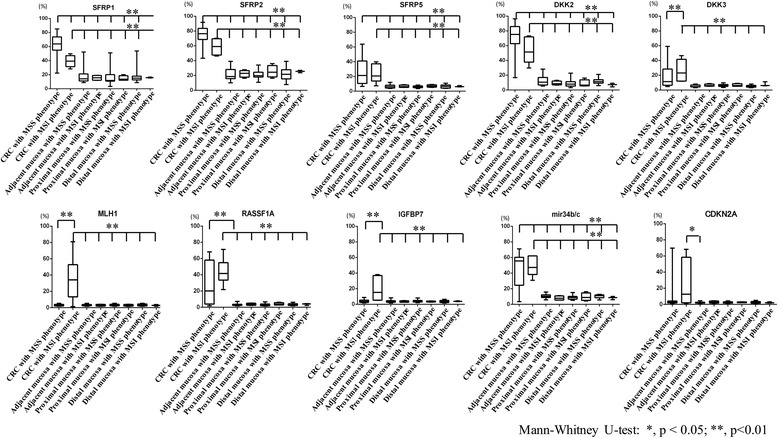
DNA methylation levels of *SFRP1*, *SFRP2*, *SFRP5*, *DKK2*, *DKK3*, *mir34b/c*, *RASSF1A*, *IGFBP7*, *CDKN2A*, and *MLH1* in CRCs with an MSS phenotype compared with CRCs with an MSI phenotype on right side CRCs depending on location relative to tumor. *p < 0.05; **p < 0.01

We also examined differences in DNA methylation between cancerous crypts from CRC and normal crypts isolated from the surrounding normal mucosa based on MSS and MSI status. The methylation levels of *SFRP1*, *SFRP2*, *SFRP5*, *DKK2*, *DKK3*, *mir34b/c*, and *RASSF1A* from MSS or MSI CRCs crypts were significantly different from those in normal crypts isolated from the surrounding normal mucosa, irrespective of sampling sites. Moreover, methylation levels of *MLH1*, *IGFBP7*, and *CDKN2A* were significantly higher in cancerous crypts isolated from CRC with the MSI phenotype than in those of the surrounding normal mucosa. There were no differences in the DNA methylation levels at the sampling sites in CRC with MSS and MSI phenotypes. The findings are shown in Fig. 2.

### Comparison of DNA methylation levels in cancer crypts with those of the surrounding normal crypts in MSS CRC

**Fig. 3:**
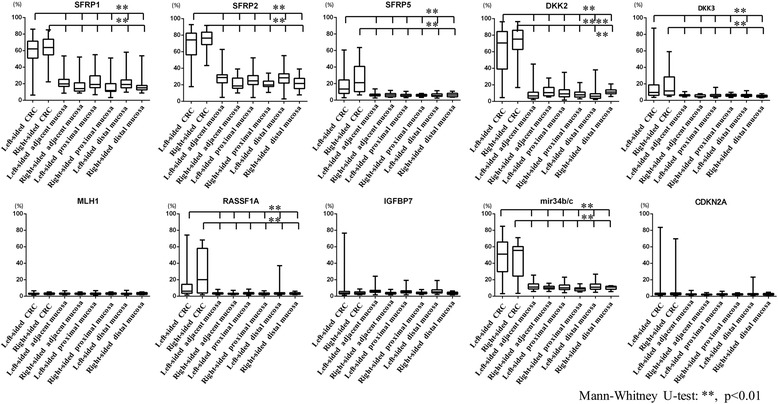
DNA methylation levels in *SFRP1*, *SFRP2*, *SFRP5*, *DKK2*, *DKK3*, *mir34b/c*, *RASSF1A*, *IGFBP7*, *CDKN2A*, and *MLH1* in CRCs with an MSS phenotype on the left side compared with the right-side CRCs with an MSS phenotype depending on the location relative to the tumor **p < 0.01

We examined DNA methylation levels of *SFRP1*, *SFRP2*, *SFRP5*, *DKK2*, *DKK3*, *mir34b/c*, *RASSF1A*, *IGFBP7*, *CDKN2A*, and *MLH1* genes in cancerous crypts in CRC. We also examined the MSS phenotypes and the tumor location (left versus right side). There were no significant differences in the DNA methylation levels between crypts from the left versus the right side in MSS CRC. Compared to surrounding normal colonic crypts, the methylation levels of *SFRP1*, *SFRP2*, *SFRP5*, *DKK2*, *DKK3*, *mir34b/c*, and *RASSF1A* in MSS CRCs were significantly higher in cancer crypts. This was observed on both sides. The differences of DNA methylation levels of *IGFBP7*, *MLH1*, and *CDKN2A* were not significant. In addition, there were no significant differences of DNA methylation levels in the examined markers among the normal colonic crypts in the left side versus the right side of MSS CRCs. The findings are depicted in Fig. 3.

### Analysis of DNA methylation levels in surrounding normal mucosa of MSS CRCs on the left and right sides

**Fig. 4:**
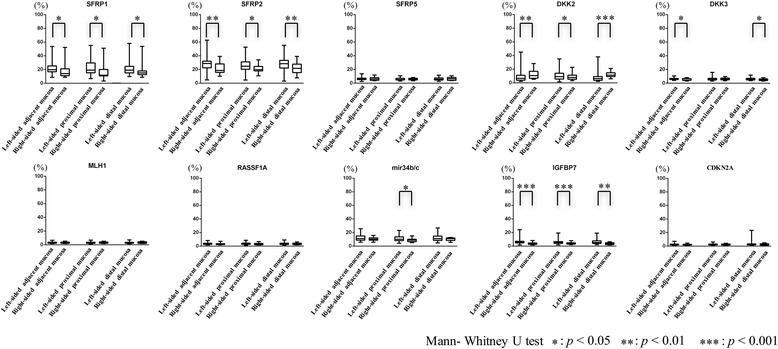
DNA methylation levels in *SFRP1*, *SFRP2*, *SFRP5*, *DKK2*, *DKK3*, *mir34b/c*, *RASSF1A*, *IGFBP7*, *CDKN2A*, and *MLH1* of normal crypts in the left-side MSS phenotype CRCs compared to the right-side CRCs with MSS phenotype depending on the location relative to the tumor. **p < 0.01; ***p < 0.001

We examined the DNA methylation levels of *SFRP1*, *SFRP2*, *SFRP5*, *DKK2*, *DKK3*, *mir34b/c*, *RASSF1A*, *IGFBP7*, *CDKN2A*, and *MLH1* in normal colonic crypts isolated from regions that were adjacent to the cancer, as well as distal and proximal regions (left and right sides). DNA methylation levels of *SFRP1* and *SFRP2* of normal crypts from regions adjacent to the cancer, distal and proximal regions were significantly higher on the left side of the colon than the right side. Whereas DNA methylation levels of *DKK2* of normal crypts from regions adjacent to the cancer and distal region were significantly higher on the right side than the left side of the colon, that from the proximal region was significantly higher on the left side of the colon than the right side. On the other hand, the DNA methylation levels of *DKK3* in normal crypts from regions adjacent to the cancer and distal regions on the left side of the colon were significantly higher than those on the right side. There was a significant difference in the DNA methylation frequencies of the *mir34b/c* gene in normal crypts from the proximal region between the left and right sides of the colon. In addition, we found significant differences in the DNA methylation levels of *IGFBP7* of normal crypts from the region adjacent to the cancer and proximal and distal regions between the left- and right-side colonic mucosa. Finally, there were no differences in the DNA methylation levels of *SFRP5*, *MLH1*, *RASSF1A*, and *CDKN2A* of normal crypts from regions adjacent to the cancer, proximal and distal regions between the left and right sides of the normal colons. These findings are shown in Fig. 4.

### DNA methylation of *MLH1* and *CDKN2A:* validation based on their expression

We examined the association between DNA methylation levels of two of the genes in this study and their expression status using immunohistochemical staining (IHC). We examined four tumors with positive *MLH1* methylation and 10 tumors with negative *MLH1* methylation. Here, positive DNA methylation of *MLH1* was defined as more than 30% methylation. Whereas loss of MLH1 expression was found in four of four *MLH1*-positive methylations, expression of MLH1 was seen in 10 of 10 *MLH1*-negative methylations. In addition, we examined the correlation of *CDKN2A* methylation and expression (eight tumors with positive methylation of *CDKN2A* and five tumors with negative methylation of CDKN2A). Positive DNA methylation of *CDKN2A* was defined as a level greater than 30%. Thus, loss of expression of CDKN2A occurred in six of eight CRCs with *CDKN2A* methylation. On the other hand, expression of CDKN2A was found in five of five CRCs without methylation of *CDKN2A*. Thus, there were significant associations of DNA methylation of *MLH1* and *CDKN2A* with expression of MLH1 and CDKN2A, as shown in Additional file 2: Table S2. Unfortunately, antibodies against the remaining genes we examined were not available.

### Analysis of sensitivity and specificity of cancer-related genes

Sensitivity and specificity were determined by the cutoff value. The optimal cutoff value for the methylation level of cancer-related genes can be defined by receiver operating characteristic (ROC) analysis of sensitivity and specificity at different cutoff values. The sensitivity and specificity determined by the cutoff values are shown in Additional file 3: Table S3. *SFRP1*, *SFRP2*, and *DKK2* were appropriate markers to differentiate colorectal cancer cells from normal crypt cells.

### Mutations of *KRAS* and *BRAF* genes in CRC

Mutations of *KRAS* and *BRAF* genes were examined to identify genetic characteristics of the CRCs. Mutations of *KRAS* and *BRAF* genes were found in 28 and 6 out of 100 CRCs, respectively. Although mutations of the *KRAS* gene were seen in CRCs with an MSS phenotype (94 CRCs), mutations of the *BRAF* gene were observed in 5 of 6 CRCs with an MSI-high phenotype.

## Discussion

Aberrant DNA methylation is a key alteration in colorectal carcinogenesis, and a large number of genes that are targets of aberrant methylation have been reported [6–8, 31]. In the present study, *SFRP1*, *SFRP2*, *SFRP5*, *DKK2*, *DKK3*, *mir34b/c*, *RASSF1A*, *IGFBP7*, *CDKN2A*, and *MLH1* were examined for DNA methylation status in isolated cancerous crypts from CRC and the surrounding normal crypts. In addition, the DNA methylation status was quantified as a methylation level using a combination of pyrosequencing methods and crypt isolation that obtained purified tumor or normal crypts. We selected 10 markers to examine DNA methylation level based on microsatellite phenotype. The reasons are as follows: (1) DNA methylation of *SFRP1*, *SFRP2*, *SFRP5*, *DKK2*, *DKK3*, and *RASSF1A* inhibits Wnt signaling that may play an important role in CRCs with both phenotypes of MSS and MSI and (2) *IGFBP7*, *CDKN2A*, and *MLH1* are specifically methylated in right-side CRCs with an MSI phenotype. In addition, *mir34b/c* is commonly methylated in both phenotypes of CRCs. Therefore, we selected these markers to elucidate the role of DNA methylation in CRC and the surrounding normal mucosa during colorectal carcinogenesis. Next, it is well known that DNA methylation is related to pathway-specific predisposition to cancer and/or tumor location in CRC [12, 29]. We attempted to identify the role of DNA methylation in cancerous crypts and the normal crypts isolated from CRC and the surrounding mucosa using several cancer-related markers.

In the present study, we examined *KRAS* and *BRAF* mutations to define the genetic profile of the CRCs we examined. Whereas mutations of *KRAS* were found in CRCs with an MSS phenotype, mutations of *BRAF* were seen only in CRCs with an MSI phenotype. We suggest that this genetic profile of *KRAS* and *BRAF* mutations in the present study did not bias the analysis of CRCs that we examined.

We examined left- and right-side CRCs with an MSS phenotype to determine if relevant genes differed in DNA methylation levels. Thus, we surveyed *SFRP1*, *SFRP2*, *SFRP5*, *DKK2*, *DKK3*, *mir34b/c*, *RASSF1A*, *IGFBP7*, *CDKN2A*, and *MLH1*. In the present study, the left- and right-side CRCs with an MSS phenotype did not significantly differ in DNA methylation levels in the examined markers except for *SFRP5*. Recent study has shown that although there is a significant difference of copy number alterations between left- and right-side CRCs with an MSS phenotype, no difference of DNA methylation status between them was found using a two-panel method that assessed genome-wide methylation status [12]. We suggest that there is no epigenetic difference of the examined markers between the left- and right-side CRC with an MSS phenotype.

It is well recognized that a CRC with an MSS phenotype is quite different from an MSI phenotype in terms of clinicopathological and molecular findings [3, 5]. In the present study, we examined MSS and MSI phenotypes to determine the differences in DNA methylation levels on the right-side CRCs for the markers examined. Contrary to expectations, DNA methylation levels in *SFRP1*, *SFRP2*, *DKK2*, and *mir34b/c* genes were higher in MSS CRC than in MSI CRC in the right-side CRCs. This finding suggests that DNA methylation occurring in some cancer-related gene (e.g., *SFRP1*, *SFRP2*, *DKK2*, and *mir34b/c*) plays important roles in the development of right-side CRCs with MSS phenotype, rather than that of MSI phenotype.

In previous studies of CRC, DNA methylation was more closely associated with an MSI phenotype than an MSS phenotype [3, 5, 7]. However, it remains unknown how mucosal DNA methylation varied between colorectal regions and the presence of molecular pathway-specific pathology. In the present study, the frequency of *MLH1* methylation was at a low level in both normal crypts isolated from an MSS phenotype as well as those with an MSI phenotype. This finding is consistent with a previous study that found that the methylation of *MLH1* was cancer-specific and was not observed in normal colonic mucosa [29]. However, contrasting data proposed that the spread of methylation in the *MLH1* promoter in the normal colonic mucosa is closely associated with age and the development of sporadic MSI-positive colorectal cancers [35]. Although the reason for the different results is not apparent, the differences might be attributed to the fact that different CpG sites were studied [35]. Previous studies have been conducted using primers containing the CpG sites used in the present study [33, 36]. We suggest that DNA methylation within the normal colorectal mucosa is not associated with molecular pathway-specific predisposition to cancer.

There is a clear difference between CRCs that form on the right side compared with those on the left side [12, 13]. Previous study has shown that the position of the primary tumor could influence treatment choice [37]. Survival was significantly longer for patients with primary tumors that originated on the left side of the colon than for patients with primary tumors that originated on the right side of the colon [37]. These observations evoke the notion that there are molecular differences, including DNA methylation, between the left and right segments of the colon. In the present study, we examined DNA methylation levels of cancer-related genes in the normal crypts between the left and right sides, with a focus on CRC with an MSS phenotype. Few studies have focused on this difference [12]. No differences of DNA methylation in given markers were found in normal crypts obtained between the left- and right-side CRCs in the present study. A previous study reported that specific cancer-related genes demonstrate differential methylation depending on colon location [13]. However, our findings suggest that DNA methylation of cancer-related genes plays a minor role in tumor development in normal crypts surrounding cancer.

A previous study of normal mucosa surrounding cancer tissue showed that methylation decreased with increasing distance from the tumor, a proposed indicator of a “field effect” in CRC [18]. Shen et al. observed that the DNA repair gene O^6^-methylguanine-DNA methyltransferase (*MGMT*) was hypermethylated and silenced in colorectal tumors, as well as in the surrounding mucosa, suggesting the field effect of methylation in CRC [38]. In another study, *MLH1* methylation was present in adjacent normal mucosa [39]. These findings may support the concept of a “field effect” in CRCs. However, these data are not rigorous given that DNA methylation levels of cancer-related genes of normal crypts cannot be correlated with their distance from the CRC.

Here, the DNA methylation levels of *SFRP1* and *SFRP2* in normal crypts at various sampling sites were significantly higher in the left-side CRC with an MSS phenotype than the right-side CRC. There were significant differences of DNA methylation levels in *DKK3* and *IGFBP7* genes between various samples in the left side versus the right side. However, the DNA methylation levels of such genes were at a low level. This finding showed that normal crypts surrounding CRC increase the DNA methylation level of cancer-related genes, suggesting increasing oncogenic status of the surrounding normal crypts. Finally, our results are consistent with a previous study reported by Patai et al. [40]. Both suggest that the hypermethylated *SFRP1* gene might be useful for the early detection of CRC. In addition, our results suggest that *SFRP1*, *SFRP2*, and *DKK2* markers can differentiate colorectal cancer cells from normal crypt cells. We believe that our study provides novel findings that help to evaluate colorectal carcinogenesis.

## Conclusions

We examined DNA methylation levels of *SFRP1*, *SFRP2*, *SFRP5*, *DKK2*, *DKK3*, *mir34b/c*, *RASSF1A*, *IGFBP7*, *CDKN2A*, and *MLH1* in cancerous glands and normal crypts isolated from cancer tissue and the surrounding normal mucosa. DNA methylation levels of *SFRP1*, *SFRP2*, *DKK2*, and *mir34b/c* were associated with the development of CRCs with an MSS phenotype. The normal crypts surrounding cancer tissue did not show evidence of a decreasing gradient of methylation with increasing distance from the tumor, irrespective of a tumor’s microsatellite status and tumor location. The DNA methylation frequencies of *SFRP1* and *SFRP2* genes in normal crypts isolated from various sampling sites occurred at a high level in the left-side CRC with an MSS phenotype. Our results will need to be considered in future studies of DNA methylation in the normal colon that might be related to the presence or risk of developing CRC.

## Additional files

Additional file 1: Table S1. Primer sequences used in this study.

Additional file 2: Table S2. Relationship of DNA methylation status to expression of CDKN2A and MLH1 in colorectal carcinoma.

Additional file 3: Table S3. Analysis of sensitivity and specificity of cancer-related genes.

## Abbreviations

CDKN2A: Cyclin-dependent kinase inhibitor 2A
CIMP: CpG island methylator phenotype
CIN: Chromosomal instability
CRC: Colorectal cancer
DKK: Dickkopf-related protein 1
IGFBP7: Insulin growth factor-binding protein 7
MLH1: Mut L homolog 1
MSI and MIN: Microsatellite instability
MSS: Microsatellite stable
*RASSF*: Ras association domain-containing protein
SFRP: Secreted frizzled-related protein 1
TGF-β: Tumor growth factor β
