# Supplementary material for: Analysis of the DNA methylation level of cancer-related genes in colorectal cancer and the surrounding normal mucosa
Source: Clin Epigenetics. 2017 May 18;9:55. doi: 10.1186/s13148-017-0352-4 (PMC5437595; doi:10.1186/s13148-017-0352-4)
Supplement: Supplementary file 1 — Primer sequences used in this study. [file 13148_2017_352_MOESM1_ESM.docx]

**Additional file 1: Table S1. Primer sequences used in this study**

|  |  | forward | reverse | product size |
| --- | --- | --- | --- | --- |
| methylation analysis | |  |  |  |
|  |  |  |  |  |
| *CDKN2A* | pyroseq PCR | GGTTGTTTTGGTTGGTGTTTT | Bio-ACCCTATCCCTCAAATCCTCTAAAA | 169 bp |
|  | Sequence primer | TTTTTTTGTTTGGAAAGAT |  |  |
|  | Sequence to analyze | ATYGYG |  |  |
|  |  |  |  |  |
| *DKK2* | pyroseq PCR | GGGTTTTTTGATTAATTAAGAGGAGA | Bio-TCTACAATAACTAAAAACAATCAAATAC | 179 bp |
|  | Sequence primer | TAATTAAGAGGAGAGTTAAA |  |  |
|  | Sequence to analyze | TYGTYGAGATTTYGGYG |  |  |
|  |  |  |  |  |
| *IGFBP7* | pyroseq PCR | AGGGTTYGGGGTAGGGGATTGGGGAT | Bio-AAAACCACACCCCRAAACRATAAAAACAC | 208 bp |
|  | Sequence primer | YGGGTGTTYGTTTATTTT |  |  |
|  | Sequence to analyze | TYGAYGTTAGTAGGAGYGYGYGYG |  |  |
|  |  |  |  |  |
| *miR-34b/c* | pyroseq PCR | GGTYGAGTGATTGTGGYGGGGG | Bio-CCTCCATCTTCTAAACRTCTCCCTTA | 176 bp |
|  | Sequence primer | TAATYGTTTTTGGAATTT |  |  |
|  | Sequence to analyze | YGYGGGTYGAGGGGYGGGGYGGGYGYG |  |  |
|  |  |  |  |  |
| *MLH-1* | pyroseq PCR | TTGGTATTTAAGTTGTTTAATTAATAGTTG | Bio-AAAATACCTTCAACCAATCACCTC | 119 bp |
|  | Sequence primer | AGTTATAGTTGAAGGAAGAA |  |  |
|  | Sequence to analyze | YGTGAGTAYG |  |  |
|  |  |  |  |  |
| *SFRP1* | pyroseq PCR | GTTTTGTTTTTTAAGGGGTGTTGAG | Bio-CTCCRAAAACTACAAAACTAAAATAC | 202 bp |
|  | Sequence primer | GYGTTTGGTTTTAGTAAAT |  |  |
|  | Sequence to analyze | TTGYGYGGGGYGGTTTYGAGGGTTYG |  |  |
|  |  |  |  |  |
| *SFRP2* | pyroseq PCR | AATTTYGGATTGGGGTAAAATAAGTT | Bio-TTAAACAACAAACAAAAAAACCTAACC | 182 bp |
|  | Sequence primer | YGTTTTYGTTAGTATTTGG |  |  |
|  | Sequence to analyze | TYGYGAGGTYGTTYGYG |  |  |
|  |  |  |  |  |
| *RASSF1* | pyroseq PCR | GAAGGAGGGAAGGAAGGGTAAG | Bio-RCCTCCCCCAAAATCCAA | 148 bp |
|  | Sequence primer | TTGTATTTAGGTTTTTATTG |  |  |
|  | Sequence to analyze | CGCG |  |  |
|  |  |  |  |  |
| *DKK3* | pyroseq PCR | GATTTTGTTGAGTTTAGTTTTTTTTGGT | Bio-CAAACCTCTCTCAACCCCTACCTA | 123bp |
|  | Sequence primer | TTTTTTGGTGGATGTG |  |  |
|  | Sequence to analyze | GGGYGGGGYGTTYGAGTAGGATTYGAYG |  |  |
|  |  |  |  |  |
| *SFRP5* | pyroseq PCR | AAATGTTTAGGGAGGTAGGGAGTTT | Bio- ATAAAAACGACCCTCAACCCCTCC | 149 bp |
|  | Sequence primer | GGAGTTTTGGGGAGAA |  |  |
|  | Sequence to analyze | AYGTTGGGYGAGGTTAGGGTTGYG |  |  |
