# Supplementary material for: Analysis of the DNA methylation level of cancer-related genes in colorectal cancer and the surrounding normal mucosa
Source: Clin Epigenetics. 2017 May 18;9:55. doi: 10.1186/s13148-017-0352-4 (PMC5437595; doi:10.1186/s13148-017-0352-4)
Supplement: Supplementary file 2 — Relationship of DNA methylation status to expression of CDKN2A and MLH1 in colorectal carcinoma. [file 13148_2017_352_MOESM2_ESM.docx]

**Additional file 2: Table S2: Relationship of DNA methylation status to expression of CDKN2A and MLH1** **in colorectal carcinoma**

|  |  | *CDKN2A* methylation | | p-value |
| --- | --- | --- | --- | --- |
|  |  | (-) | (+) |  |
| CDKN2A expression | (-) | 0 | 6 | p=0.02098 |
|  | (+) | 5 | 2 |  |
|  |  |  |  |  |
|  |  | *MLH-1* methylation | | p-value |
|  |  | (-) | (+) |  |
| MLH-1 expression | (-) | 0 | 4 | p=0.000999 |
|  | (+) | 10 | 0 |  |
