# Supplementary material for: Analysis of the DNA methylation level of cancer-related genes in colorectal cancer and the surrounding normal mucosa
Source: Clin Epigenetics. 2017 May 18;9:55. doi: 10.1186/s13148-017-0352-4 (PMC5437595; doi:10.1186/s13148-017-0352-4)
Supplement: Supplementary file 3 — Analysis of sensitivity and specificity of cancer-related genes. [file 13148_2017_352_MOESM3_ESM.docx]

**Additional file 3: Table S3: Analysis of sensitivity and specificity of cancer –related genes**

|  | Cutoff value (%) | Sensitivity | Specificity |
| --- | --- | --- | --- |
| *SFRP1* | 29.8 | 0.944 | 0.889 |
| *SFRP2* | 43.7 | 0.911 | 0.956 |
| *SFRP5* | 9.9 | 0.744 | 0.957 |
| *DKK2* | 22.4 | 0.911 | 0.956 |
| *DKK3* | 8.8 | 0.611 | 0.957 |
| *RASSF1A* | 5.7 | 0.667 | 0.921 |
| *mir34b/c* | 6.7 | 0.856 | 0.889 |
| *CDKN2A* | 5.2 | 0.239 | 0.967 |
| *MLH-1* | 4.1 | 0.244 | 0.854 |
